# Comparative Performance of Bio-Carbon and Petroleum Coke as Reductants in CaCl_2_ Assisted Direct Reduction of Chromite

**DOI:** 10.3390/ma19183840

**Published:** 2026-09-09

**Authors:** David Carter, Jason P. Coumans, Nail Zagrtdenov, Dominique Duguay, Dogan Paktunc

**Affiliations:** CanmetMINING, Canadian Centre for Mineral and Energy Technology, Natural Resources Canada, Ottawa, ON K1A 0G1, Canadadominique.duguay@nrcan-rncan.gc.ca (D.D.); dogan.paktunc@kromtech.ca (D.P.)

**Keywords:** ferrochrome, bio-carbon, chromite, carbothermic reduction, green metallurgy

## Abstract

CaCl_2_-assisted Direct Reduction of Chromite (DRC) is a potentially lower energy alternative to conventional chromite smelting to produce ferrochrome (FeCr). This study investigates the technical feasibility of substituting petroleum coke (PC) with bio-carbon (BC) as the reductant in DRC and quantifies its effects on reduction behavior, alloy characteristics, and residual phase evolution. Chromite pellets containing CaCl_2_ flux and either PC or BC were reduced under controlled conditions using thermogravimetric analysis and electric tube furnace experiments with continuous off-gas monitoring. Products were characterized using various methods including 3D X-ray microtomography, Scanning Electron Microscope (SEM)-based quantitative mineralogy, and Wavelength Dispersive Spectrometry using an Electron Probe Microanalyzer (WDS-EPMA). BC accelerated reduction kinetics relative to PC due to its devolatilization, which generated a microporous network and increased the reactive surface area. Correspondingly, peak CO flux occurred 8 min earlier with BC during vertical tube furnace tests. BC also produced finer FeCr alloys, with 15% of alloy volume below the initial reductant particle size compared with 3% for PC, reflecting enhanced alloy densification. Alloy compositions using both reductants met high-carbon FeCr specifications. BC use also promoted the formation of non-olivine and Cl-bearing slag phases, indicating that reductant type can influence the partitioning behavior of non-alloying elements within slag phases.

## 1. Introduction

Chromium is a critical alloying element in stainless steel, providing strength, toughness, and corrosion resistance. During stainless steel making, chromium is introduced as ferrochrome (FeCr), which is conventionally produced by the smelting of chromite ore with fluxes and carbonaceous reductants in AC or DC Arc furnaces. This process is highly energy and carbon intensive due the substantial latent heat of fusion required for smelting. Conventional FeCr production consumes 3.8–4.8 MWh/tFeCr and emits 5.3–5.5 tCO_2_e/tFeCr [1,2,3].

Efforts to reduce these impacts have led to the development and commercialization of the Premus process (Glencore) and Oxidative Sintered Pelletized Feed process (Outotec). The Premus process achieves energy savings by pre-reducing Fe and Cr in a rotary kiln prior to smelting [4], while the oxidative sintered pelletized feed process enhances chromium reducibility by generating shear forces during the magnetite to hematite transformation during oxidation [3,5]. These innovations reduce Specific Energy Consumption (SEC) and carbon intensity to 2.4 MWh/t FeCr and 4.8 tCO_2_e/tFeCr (Premus–Glencore) and 3.1 MWh/t FeCr and 2.2 tCO_2_e/tFeCr (Oxidative sintered pellet–Outotec) [1,6,7,8]. However, smelting still accounts for 64–98% of the total energy use and 69–99% of greenhouse gas emissions [1], primarily from charge coke used for carbothermic reduction. To reduce the carbon intensity of conventional FeCr production, up to 25% of this charge coke could potentially be replaced with bio-carbon (BC) from pyrolyzed biomass. However, complete substitution remains challenging due to differences in mechanical strength, density, porosity and CO_2_ reactivity [9,10,11]. Additionally, BC generally exhibits higher CO_2_ reactivity, increasing reductant consumption via the Boudouard reaction, which does not contribute to chromium reduction. Although processing conditions can improve BC properties, cokes remain the preferred reductant for metallurgical applications [9,11,12].

Alternatively, renewable H_2_ could potentially be used to lower the carbon intensity of FeCr production. Recent laboratory studies have shown that Fe-oxides in chromite can be metallized using H_2_ [13,14,15], whereas the more stable Cr oxides remain largely unreduced. As a result, subsequent carbothermic reduction is required to recover Cr, increasing process complexity but potentially reducing carbon emissions by 16% [14].

The 2008 discovery of Ontario’s Ring of Fire chromite deposits provides Canada with a unique opportunity to develop its chromite industry using new FeCr processing technologies with lower SEC and carbon intensity, informed by recent research [16,17,18]. The CaCl_2_ flux-assisted Direct Reduction of Chromite (DRC) process is particularly promising. This method involves the dissolution of Fe and Cr oxides into molten CaCl_2_ and slag, followed by reduction at carbonaceous particle surfaces via a shrinking-core mechanism [18,19,20,21]. The DRC process thus yields alloy and slag products, along with residual chromite and reductant particles depending on the operating conditions. CaCl_2_ is removed from the solid products by water leaching and recovered for reuse through evaporation of the leachate. Typical CaCl_2_ recoveries exceed 55% [18]. The slag products are dominated by spinel, but also includes meaningful amounts of olivines, silicates, and glassy interstitial phases. Minor amounts of other oxides, carbonates, and sulfides may also be present. Thus, the slag produced from DRC can be considered as a salable byproduct with potential as a refractory raw material [18,19,20,21,22,23,24]. Unlike conventional smelting, the DRC process does not require high-strength coke, making complete substitution with BC technically feasible.

This study investigates the performance of BC as a reductant in CaCl_2_-assisted DRC, comparing reduction behavior, ferrochrome quality, slag and residual phase chemistry to those obtained with petroleum coke (PC). While the concept of utilizing BC was introduced by Yu and Paktunc in 2018 [19], its practical feasibility remains largely unexplored. Although BC performance in the DRC process could be further optimized, this work addresses a critical knowledge gap regarding PC substitution with BC and supports the development of low-emission FeCr production technologies.

## 2. Materials and Methods

### 2.1. Materials

**Figure 1:**
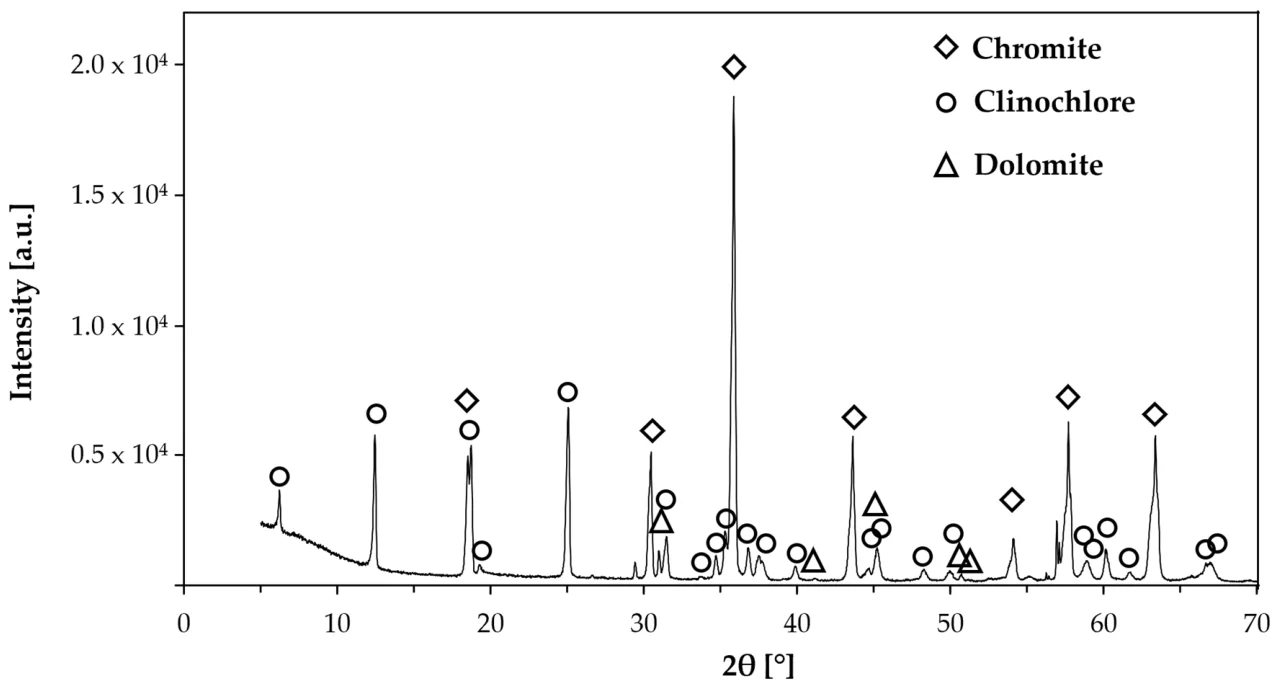
XRD Pattern of the chromite ore.

Chromite ore from the Ring of Fire deposits (Ontario, Canada) was used in all experiments. The ore was milled and sieved to a particle size range of −106 + 38 μm. Its chemical composition, determined by X-Ray Fluorescence (XRF) at Actlabs (Ancaster, ON, Canada), is shown in Table 1. X-Ray Diffraction (XRD) analysis (Figure 1) shows that the ore consists of chromite, clinochlore, and minor dolomite. A more detailed characterization of this ore is provided in previous works [19,25].

Two carbonaceous reductants were used in this work: calcined petroleum coke (PC, grade 4079, Asbury, NJ, USA) and a commercial lump charcoal (BC). The BC was produced from oak and hickory feedstocks. Proximate analysis, conducted according to ASTM D7582 [26], determined its moisture and ash contents to be 5.17 wt% and 3.62 wt%, respectively. The densities of PC and BC were measured by gas-displacement pycnometry and were 2.02 and 1.60 g·cm^−3^, respectively. Both reductants were ground and sieved to −106 + 75 µm (or −75 µm for BC where specified) following established procedures [18,21]. Proximate and ultimate analyses were performed at CanmetENERGY (Ottawa, ON, Canada) and are shown in Table 2. Reductant proportions relative to 100 parts ore are 18 for PC and 15 to 30 for BC.

Anhydrous CaCl_2_ was used as the flux in all experiments at a fixed ore:flux mass ratio of 100:30.

### 2.2. Furnace Tests

Pellets were prepared using mixtures of ore, reductant (PC or BC) and CaCl_2_ as flux in the proportions shown in Table 3. These compositions were informed by previous works to investigate the listed design considerations [18,21]. Experimental design conditions included various proportions of BC as a reductant that include sub-stoichiometric and super-stoichiometric fixed carbon as needed for the complete reduction of Fe as FeO and Cr as Cr_2_O_3_ to (Fe,Cr)_7_C_3_ + CO [18]. Smaller particle sizes were investigated in the TG2 test to ensure that any residual chromite after reduction is attributable to a C deficiency and to determine the optimum proportion of BC. ET3 tests used larger sample masses and incorporated the theoretical reduction equivalent (relative to PC) of BC required for the reduction of Fe and Cr as determined in TG2 tests, alongside the optimized PC amount identified in previous studies [18,21]. This methodology provided a representative basis for comparing BC and PC as reductants in terms of reduction behavior, alloy quality, and slag phase chemistry. Overall, these tests were designed to address the objectives of the study in assessing the performance of BC as a reductant with the details listed in Table 3.

#### 2.2.1. Thermogravimetric Tests

Thermogravimetric (TG) experiments were performed using a Netzsch STA 449 F5 Simultaneous Thermal Analyzer (Netzsch Gerätebau GmbH, Selb, Germany) equipped with a differential thermal analysis sample carrier and coupled to a Hiden HPR 20 EGA quadrupole Mass Spectrometer (MS, Hiden Analytical, Warrington, UK) for off-gas monitoring. Gas species concentrations were quantified as real intensities using manufacturer-specified calibration factors [30].

For each experiment, a single pellet was prepared by mixing 250 mg of ore, reductant, and flux with 6 wt% deionized water. For the TG1 experimental series, the mass ratio of ore:reductant:flux was fixed at 100:18:30, corresponding to 68, 12, and 20 wt%, respectively. This mass proportion of ore to PC is stoichiometric for the complete reduction of Fe and Cr [18]. For the TG2 series, the mass ratio of ore:reductant:flux was 100:15–30:30, corresponding to 63–69, 10–19, and 19–21 wt%, respectively. For each pellet, the damp mixture was pressed into a 6 mm spherical pellet using concave dies. A compaction force of 750 ± 50 N was applied and maintained for 1 min. Pellet diameter was between 5.5 and 6.5 mm for all pellets. Actual dried pellet masses (Table 3) vary due to moisture uptake by hygroscopic CaCl_2_ during preparation and minor losses during sample transfer and handling. Pellets were placed in lidded alumina crucibles with a pinhole vent. An empty lidded crucible served as reference. Experiments were performed under grade 5 Argon at 200 mL·min^−1^. Pellet compositions and thermal cycles are shown in the figures.

Owing to their consistent spherical geometries, TG1 explores the effect of reductant type (BC vs. PC) at constant mass, while TG2 explores the effect of systematically varying BC content.

#### 2.2.2. Electric Tube Furnace Tests

Electric tube (ET) furnace experiments were performed in a Deltech VTOSD-33 bottom loading tube furnace (Deltech, Denver, CO, USA) with off-gas monitored by a Siemens Ultramat 23 Infra-Red analyzer (IR, Siemens, Munich, Germany).

Three or four pellets were reduced simultaneously in each experiment. Each pellet was prepared by mixing 1.75 g of ore, reductant, and flux with 6 wt% deionized water. The mass ratio of ore:reductant:flux was 100:18–27.5:30 (63–68, 12–17, and 19–20 wt%, respectively). These tests used −106 + 75 µm PC and BC as reductants (ET3-PC18, ET3-BC18, and ET3-BC27.5). For each pellet, the damp mixture was pressed into a 12 mm diameter sphere or cylinder shape using dies. A compaction force of 1500 ± 50 N was applied and maintained for 2 min. The compaction force was consistent for all pellets and so pellet size varied with reductant type and proportion relative to ore. BC-containing pellets could not be formed as spheres and were pressed as cylinders. Actual dried pellet masses (Table 3) vary due to moisture uptake by hygroscopic CaCl_2_ during preparation and minor losses during sample transfer and handling. Pellets were placed in an open topped alumina crucible and reduced under grade 5 Argon at 0.5 L·min^−1^. Pellet compositions and thermal cycles are shown in the figures.

After reduction, one pellet was scanned using 3D X-ray microtomography, then halved following washing and rinsing (Section 2.3). One half was mounted and polished for microscopy analysis and the other analyzed using XRD. The remaining pellets were lightly ground, wet sieved into 106, 75, and 38 µm bound fractions, and the alloy particles were separated from residual phases by panning and elutriation [20]. Fractions greater than 38 µm were combined for assay analysis.

### 2.3. Analytical Methods

Reduced samples were washed and rinsed three times in deionized water, then dried at 200 °C for 30 min. Dried samples were mounted in epoxy and polished for microscopy analysis.

Powder X-ray diffraction (XRD) was performed on finely ground (micronized) samples using a Rigaku D/MAX 2500 rotating-anode powder diffractometer (Rikagu, Tokyo, Japan) with Cu K radiation at 40 kV, 200 mA.

Phase proportions were determined using Tescan’s Integrated Mineral Analyzer (TIMA) based on Vega-3 SEM equipped with four EDAX EDS detectors (Tescan, Brno, Czechia). The size of the acquired pixels was 3 × 3 µm. Accelerating voltage was 25 kV and absorbed current was 5 nA during the runs.

Quantitative X-ray microanalyses were made by wavelength dispersive spectrometry (WDS) using a JEOL JXA 8230 electron probe microanalyzer (WDS-EPMA, JEOL, Tokyo Japan) operated at an accelerating voltage of 20 kV and a probe current of 20 to 25 nA. Counting times ranged from 15 to 40 s for discrete phase analyses, typically with a focused beam for alloys and a defocused beam for all other phases. Reported microanalyses with two decimal points are based on 3 sigma uncertainty with a confidence level of 99.73%. Partitioning of the measured Fe between divalent and trivalent species was based on stoichiometry. Carbon concentrations of the alloy phase were determined by difference, which is consistent for M_7_C_3_ type carbides.

Bulk chemical compositions were determined by Inductively Coupled Plasma-Optical Emission Spectrometry (ICP-OES) using an Agilent 5110 VDV (Agilent, Santa Clara, CA, USA). Samples were prepared by sodium peroxide fusion followed by dissolution of the fusion cake in 20% HNO_3_ prior to analysis. Major and trace inorganic elements were quantified by ICP-OES. Quality assurance and quality control procedures included the analysis of the certified reference materials (CRMs) GCR-01, SRM 64C, SOIL-A, SOIL-B, IV-71A, and 900-EMR512, which were processed and analyzed alongside the samples. Analytical accuracy was evaluated by comparison of measured and certified values for the CRMs. Total carbon was determined with an ELTRA CS-2000 analyzer (Eltra, Temse, Belgium).

3D X-ray microtomography analysis was conducted on intact 12 mm pellets using a Zeiss Versa 610 X-Ray Microscope (Zeiss, Oberkochen, Germany). Pellets were wrapped in parafilm after furnace processing to prevent CaCl_2_ moisture re-absorption. The pellets were scanned using an approximately 15 mm field of view at 2000 pixels per tomogram, yielding a voxel side length of approximately 7 µm (isotropic). Segmentation, particle separation, and computations of volume, surface area, and mean Feret diameters were performed in Dragonfly 3D World (version 2025.1) [31,32].

## 3. Results and Discussion

### 3.1. Comparative Performance of BC and PC as Reductants in the DRC Process

**Figure 2:**
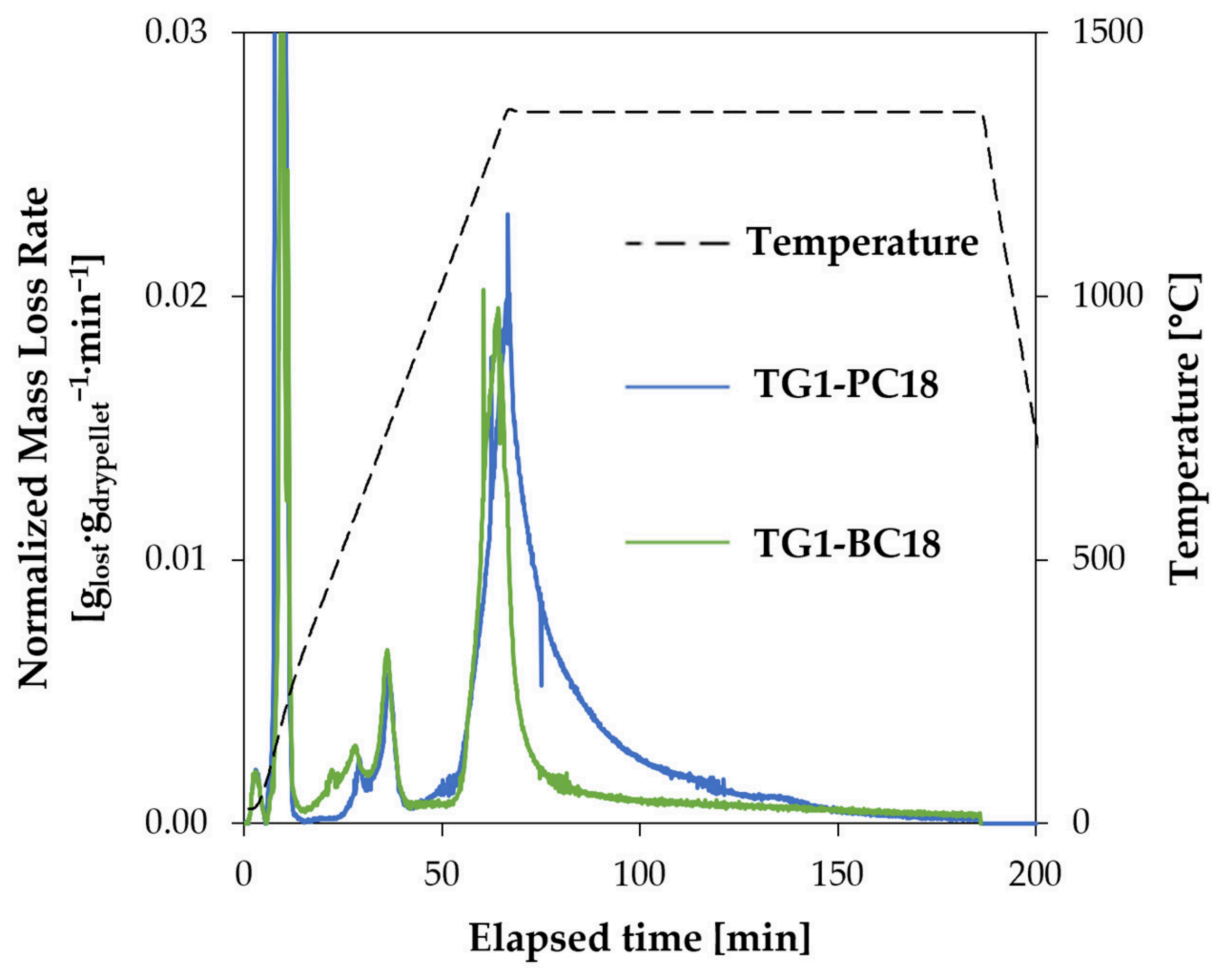
Rate of mass loss during the thermal cycle shown for pellets containing PC and BC as reductants. Mass proportion of ore:reductant:flux for both pellets is 100:18:30. Mass loss has been normalized based on the mass of dried pellets.

**Figure 3:**
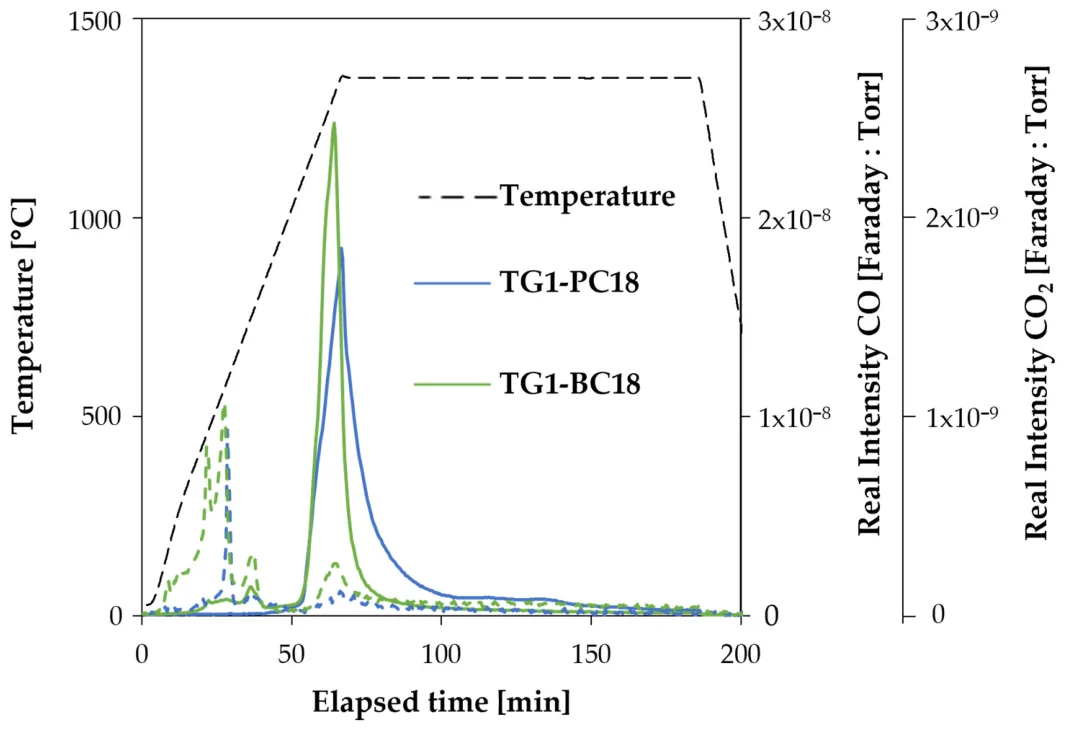
Off-gas profiles measured by MS during the thermal cycle shown for pellets containing PC and BC as reductants. Mass proportion of ore:reductant:flux for both pellets is 100:18:30. CO profiles are shown in solid lines and CO_2_ in dashed lines.

CaCl_2_-assisted DRC was first evaluated under controlled conditions using PC and BC as reductants in TGA-MS experiments (TG1). Operating conditions were determined based on previously optimized conditions using PC as a reductant [18,21]. Normalized mass loss rates and off-gas profiles are shown in Figure 2 and Figure 3, respectively.

Figure 2 and Figure 3 show that in the low-temperature regime (<14 min and <300 °C), TG1-PC18 and TG1-BC18 display similar mass loss rates which increase and decrease rapidly. Despite this similarity, measurable CO_2_ evolution is observed for TG1-BC18 but not TG1-PC18. Minor CO evolution is also detected for TG1-BC18 at approximately 12 min (257 °C), while TG1-PC18 shows no corresponding CO signal in this temperature range.

In the intermediate temperature regime between 300 and 850 °C (14–42 min), the behavior of the two samples diverges markedly. TG1-BC18 exhibits a sustained increase in mass loss rate from 300 to 465 °C (14–22 min), accompanied by clearly discernible and persistent CO_2_ evolution, whereas TG1-PC18 shows negligible mass loss and no significant CO_2_ release over the same sub-interval. From 465 to 850 °C (22–42 min), both samples undergo further mass loss. However, TG1-BC18 consistently demonstrates greater total mass loss and higher CO_2_ evolution than TG1-PC18. Within this regime, both materials exhibit a pronounced local maximum mass loss rate at approximately 36 min (741 °C). CO evolution is first detected for TG1-PC18 near the upper end of this interval, at approximately 42 min (862 °C).

At temperatures above 1050 °C (53 min), Figure 2 and Figure 3 indicate the onset of substantial mass loss accompanied by pronounced CO generation for both samples, persisting through the remainder of the isothermal dwell at 1350 °C. In this high-temperature regime, TG1-BC18 exhibits an earlier onset and a higher peak CO intensity than TG1-PC18, followed by a more rapid decline beyond approximately 65 min (1323 °C). By contrast, TG1-PC18 displays a delayed and more gradual CO evolution profile.

The data for TG1-PC18 shown in Figure 2 and Figure 3 align with previous results [18,20,21]. Evaporation of free and adsorbed water begins near 100 °C, followed by gangue mineral, clinochlore decomposition (i.e. loss of structural water) at ~500 °C, and carbonate decomposition at around 740 °C, producing CO_2_. After CaCl_2_ melts at 772 °C, carbothermic reduction of Fe begins at ~1100 °C, generating CO and accelerating with temperature. Cr reduction initiates at a higher temperature and proceeds similarly. The Boudouard equilibrium reaction (Equation (1)) proceeds simultaneously, with the forward reaction toward CO formation strongly favored at temperatures exceeding 1000 °C.

(1) $$C+{CO}_{2}↔2CO$$

TG1-BC18 contains the same mass proportion of ore as TG1-PC18 and therefore undergoes identical gangue mineral decomposition events. The ore:reductant ratio for TG1-PC18 and TG1-BC18 was additionally consistent, resulting in comparable external reductant surface areas. However, unlike PC, the BC reductant used in this work contains a higher volatile matter content (38.4% for BC vs. 0.5% for PC; Table 2) and a lower fixed carbon content (61.6% for BC vs. 99.5% for PC; Table 2). In the low-temperature regime, detectable CO_2_ evolution accompanied by minor CO release from TG1-BC18 indicates the presence and decomposition of thermally labile oxygen-containing functional groups, e.g. carboxyl groups [33,34]. In the intermediate temperature regime, TG1-BC18 exhibits sustained and greater CO_2_ evolution, indicative of additional mass loss beyond that expected from gangue mineral decomposition alone, as established by comparison with TG1-PC18. This enhanced mass loss and CO_2_ evolution are consistent with continued decomposition of functional groups such as residual carboxyl groups and more thermally stable carbonyl, ester, and lactone groups [33,34]. Early reaction progress whereby CO acts as a reductant for Fe-oxides that are not part of the chromite spinel may also occur simultaneously at temperatures above 750 °C [35,36].

The evolution of volatile matter during the low and intermediate temperature regimes generates an open, interconnected microporous network within the BC matrix [37,38,39,40]. This porous morphology increases the effective reductant surface area relative to PC, thereby accelerating initial metallization in accordance with shrinking core kinetics [18,20,21]. However, the loss of volatile matter also decreases the carbon content of BC, such that TG1-BC18 becomes sub-stoichiometric with respect to Fe and Cr reduction at higher temperatures. In contrast, PC consists predominately of fixed carbon and is not expected to undergo significant morphological change prior to the onset of carbothermic reduction. Consequently, TG1-PC18 retains its stoichiometric proportion of carbon for Fe and Cr reduction, leading to greater CO evolution at extended reaction times.

### 3.2. Influence of Bio-Carbon Proportion on Chromite Reduction

To maximize chromite reduction using BC in the CaCl_2_ assisted DRC process, sufficient carbon must remain after BC’s devolatilization for Fe and Cr reduction as discussed in Section 3.1. The BC content in DRC pellets was therefore varied to assess both reduction extent and FeCr particle morphology in TGA-MS experiments (TG2). BC size was −75 µm.

**Figure 4:**
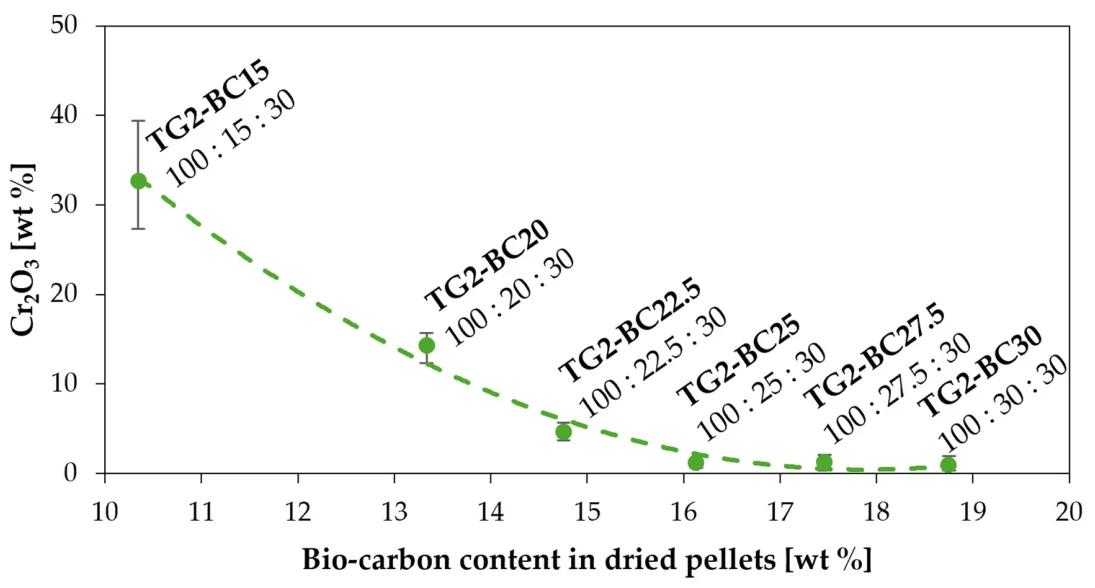
Average Cr_2_O_3_ content in residual spinel determined using WDS-EPMA analysis vs. BC content in the original green pellets. Error bars represent the range of 10 measured values.

**Figure 5:**
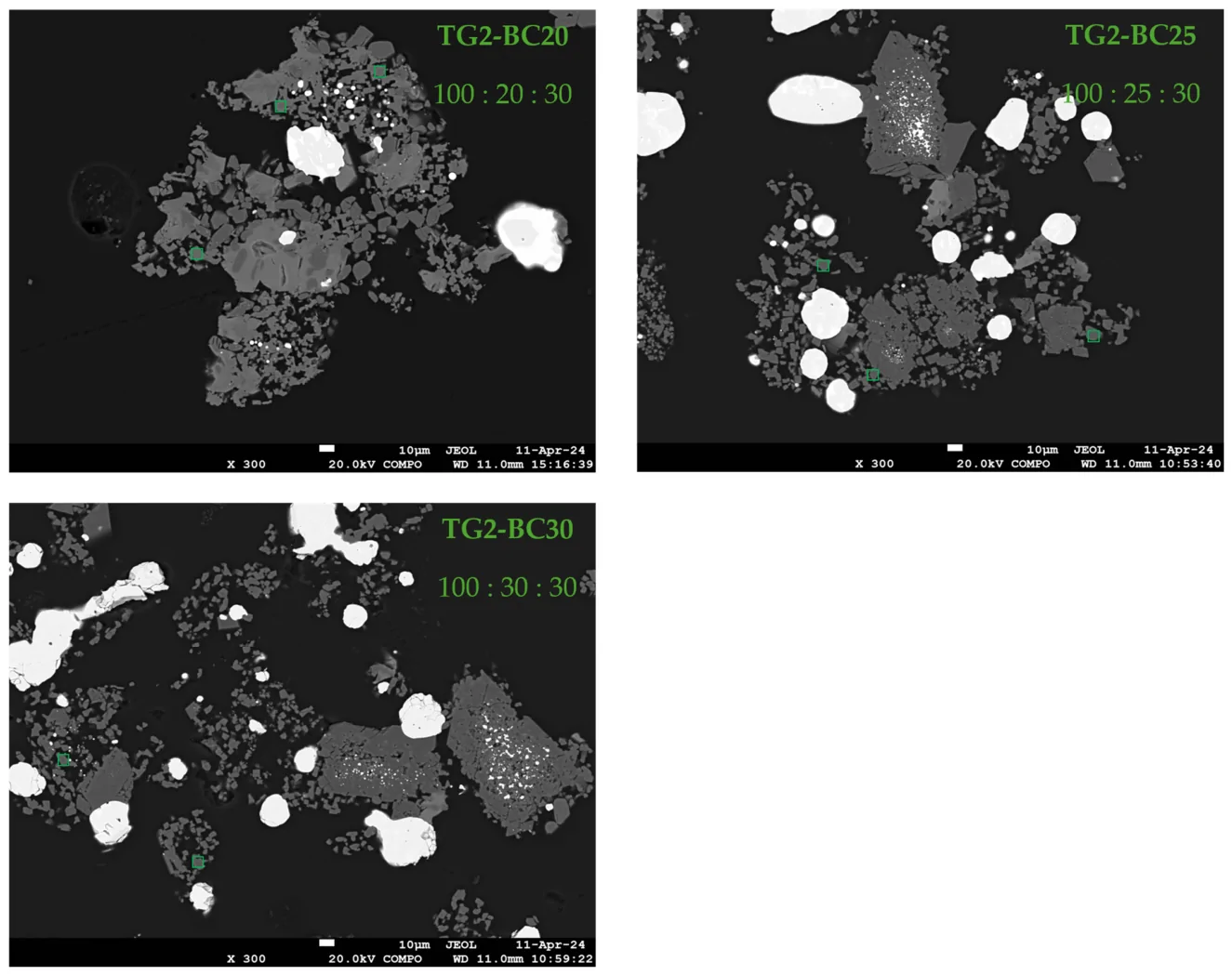
Representative BSE images of alloy and residual phases in reduced pellets. Residual phases appear in greyscale tones based on composition, while alloy particles appear white. Squares denote spinel phases analyzed using WDS-EPMA.

**Figure 6:**
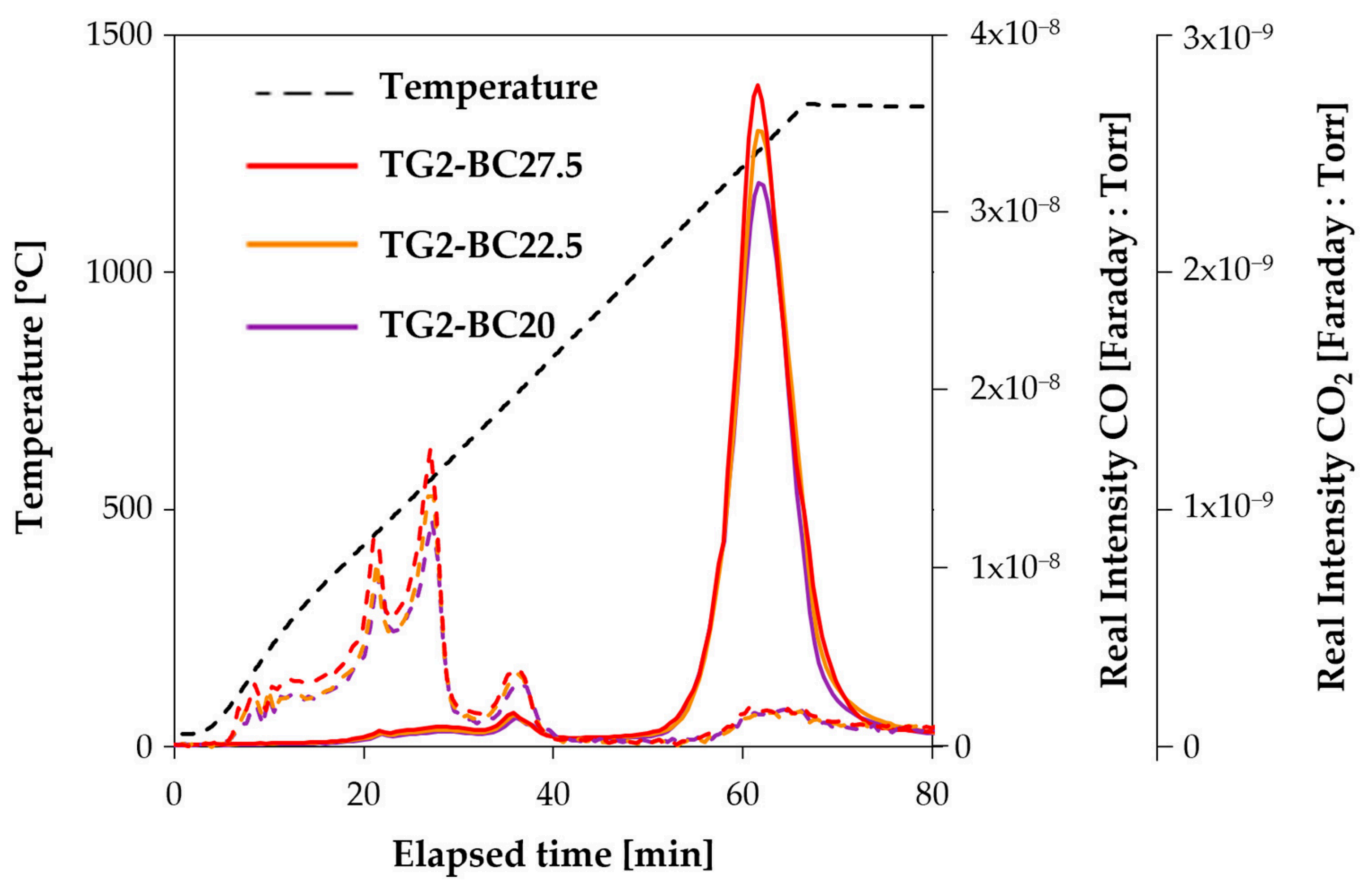
Off-gas profiles measured by MS during the peak decomposition stages of the thermal cycle shown for representative TG2 tests. TG2-BC20, TG2-BC22.2, and TG2-BC27.5 contain 100:20, 100:22.5, and 100:27.5 ore:BC respectively. CO profiles are shown in solid lines and CO_2_ in dashed lines.

Temperature was increased at a rate of 20 °C·min^−1^ up to 1350 °C, held at 1350 °C for 120 min, and decreased at 50 °C·min^−1^ to room temperature. This temperature-time profile was validated by the near zero CO concentration measured in the off-gas after approximately 120 min in TG1 experiments (Figure 3), indicating the complete consumption of reactive phases. Owing to higher surface-area-to-volume ratios and therefore faster shrinking-core reduction kinetics, the smaller BC particles in TG2 should lead to near zero CO off-gas concentrations sooner than the larger BC particles in TG1. Cr_2_O_3_ concentrations in residual spinel grains were determined using WDS-EPMA analysis as shown in Figure 4. Representative back scattered electron (BSE) images are shown in Figure 5. CO and CO_2_ off-gas profiles for TG2-BC20, TG2-22.5, and TG2-BC27.5 are shown in Figure 6.

Figure 4 shows that the Cr_2_O_3_ content in residual spinels is minimized to an average value of less than 1.27 wt% when the pellet composition includes a mass ratio of at least 100:25 ore:BC. The BSE images shown in Figure 5 all reveal the presence of sub −5 µm FeCr inclusions within the cores of residual spinel grains and larger FeCr particles external to spinel grains that do not contain unreacted carbon cores. At 100:30:30 (ore:BC:flux), adjoined FeCr particles whose sizes exceed the starting BC particle size are observed. The off-gas profiles in Figure 6 show BC decomposition events during the low and intermediate temperature regimes (up to 850 °C, 42 min) that are comparable to those of TG1-BC18, which increase with BC addition. At temperatures greater than 1050 °C, pronounced CO generation occurs, which likewise increases with BC addition. A minimal shift to earlier times in the peak CO concentration is observed with increasing BC content.

Stoichiometric amounts of carbon are required to reduce Fe and Cr oxides and to minimize the retention of Cr_2_O_3_ within spinel phases. Under carbon-excess conditions, however, FeCr particles may form with unreacted carbon cores, as observed by Yu and Paktunc when excess graphite was used as a reductant [20]. The absence of unreacted carbon cores in pellets with ore:BC mass ratios up to 100:30 (Figure 5) is therefore unexpected given the comparable minimized Cr_2_O_3_ concentration within spinel phases (suggesting carbon-excess amounts) at ore:BC mass ratios of 100:25, 100:27.5, and 100:30 ore:BC (Figure 4). Unreacted carbon cores were also observed in Yu and Paktunc’s related study, where chromite was reduced using a different BC with larger particle sizes (180–300 µm) at a comparatively lower temperature of 1300 °C, [19]. The unreacted carbon core retention in [19] is attributable to slower reaction kinetics associated with lower surface area to volume ratios of the larger reductant particles. The absence of unreacted carbon cores in the present work therefore suggests that after the complete reduction of Fe and Cr, residual BC is consumed by an additional reaction that is promoted or becomes more favorable at the comparatively higher temperature used in this study (1350 vs. 1300 °C).

The fine alloy inclusions (5 µm) within spinel grains form through CO-mediated reduction [18,19,41]. They are notably undesirable because they require intensive grinding for liberation and are difficult to recover from similarly sized residual phases. Evaluation of alloy morphologies using larger and more representative samples was investigated in the following section using X-Ray microtomography.

Figure 4 shows that TG2-BC27.5 contains the minimized Cr_2_O_3_ content. Figure 5 further indicates that its alloy morphology and absence of unreacted reductant cores will be intermediate between TG2-BC25 and TG2-BC30. The fixed carbon content of TG2-BC27.5 additionally approximates the reference ore:PC ratio of 100:18 without exceeding it. On this basis, an ore:BC ratio of 100:27.5 is considered appropriate for further investigation and comparison with tests using the previously optimized ore:PC ratio of 100:18 [18].

### 3.3. Detailed Chemical and Microstructural Comparison of Reduced Pellets Using Petcoke and Bio-Carbon as Reductants

Electric tube furnace tests with IR off-gas analysis (ET3) were conducted to generate larger sample masses and provide representative data for comparing BC and PC as reductants in terms of reduction behavior, alloy quality, and slag phase chemistry. Ore and reductant size ranges were consistent with those used in Section 3.1.

**Figure 7:**
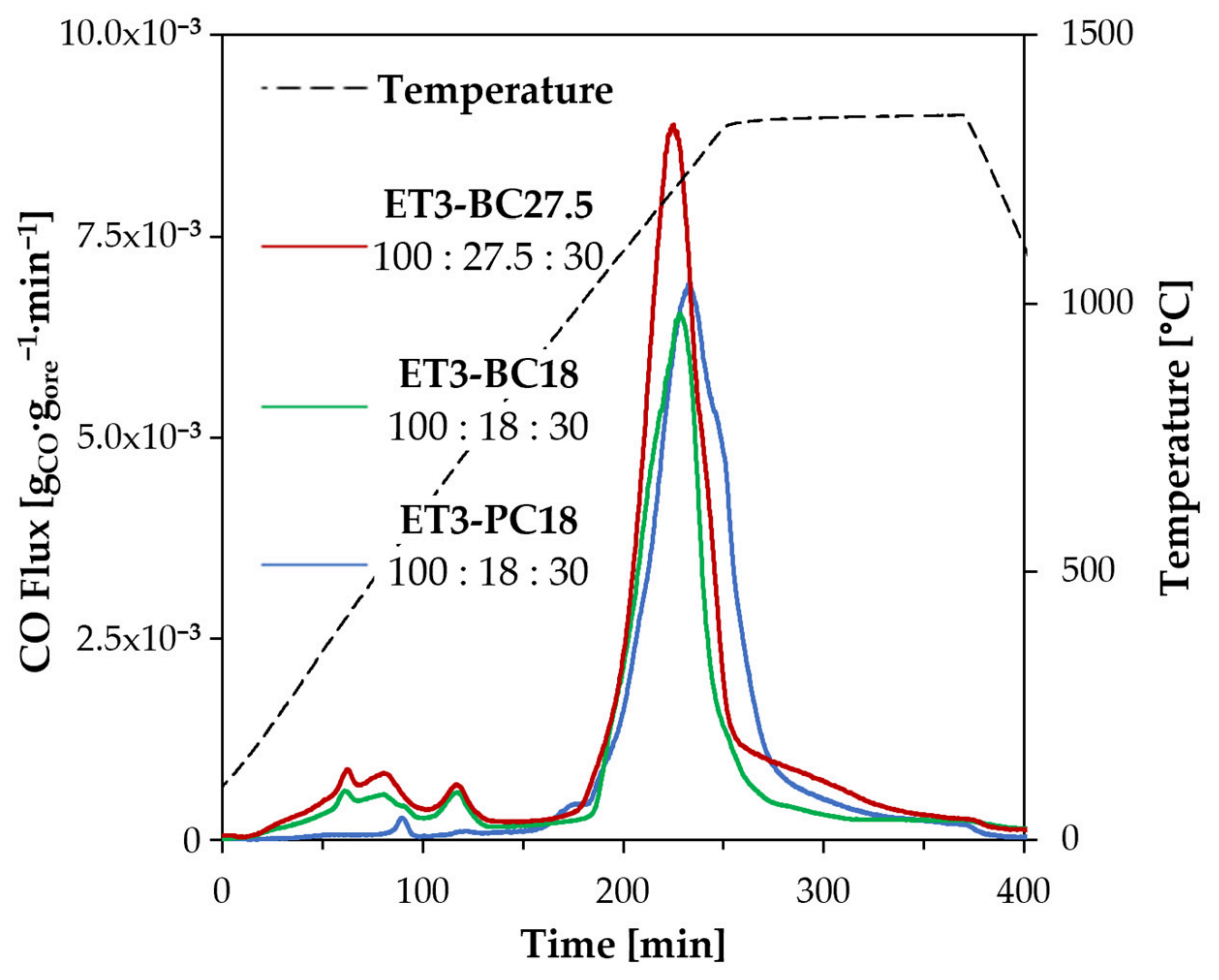
CO flux calculated from IR-measured concentrations and normalized to the initial ore mass in pellets before furnace processing. Thermal cycle is shown in dashed line.

**Figure 8:**
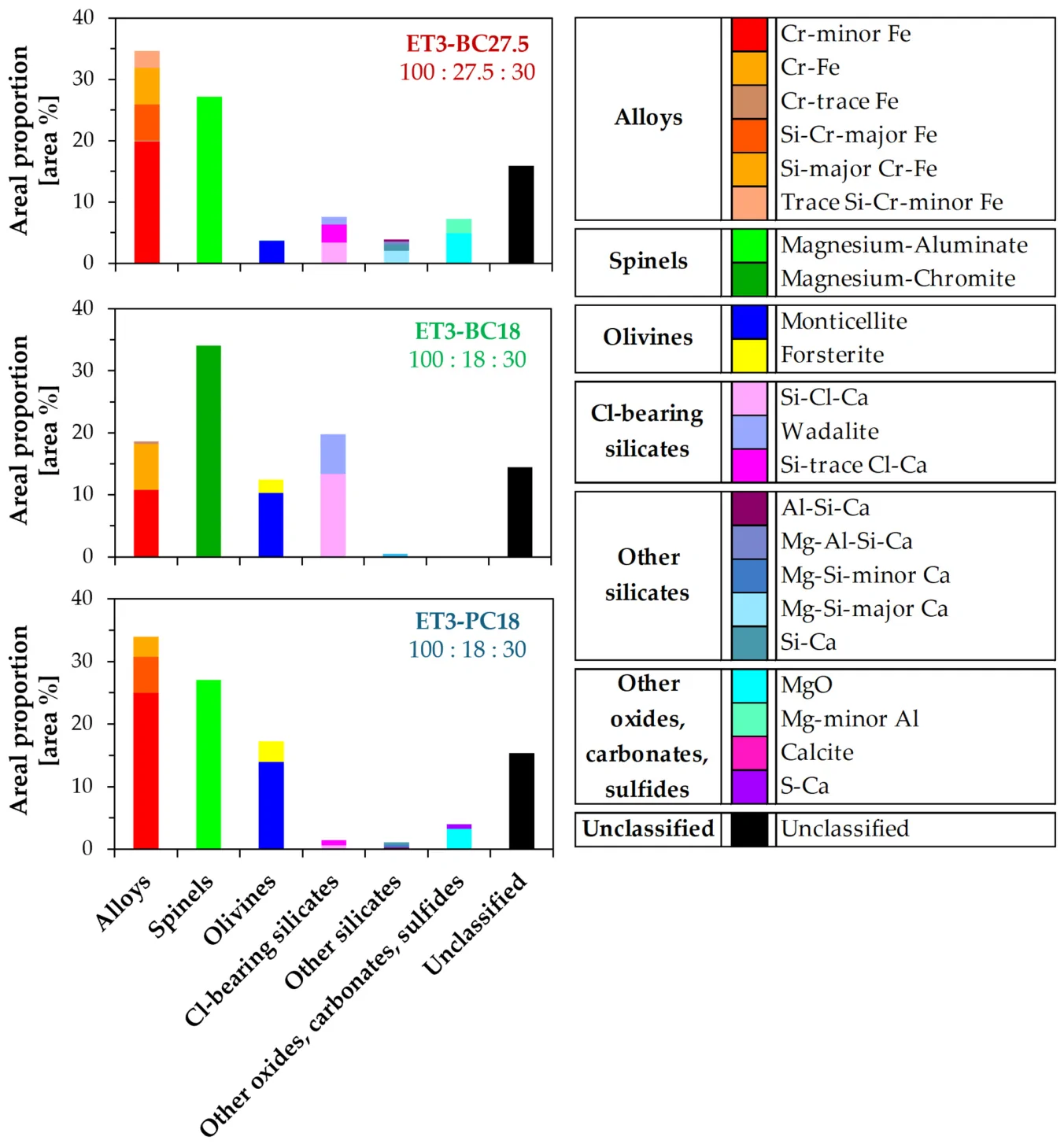
Phase proportions determined using TIMA.

**Figure 9:**
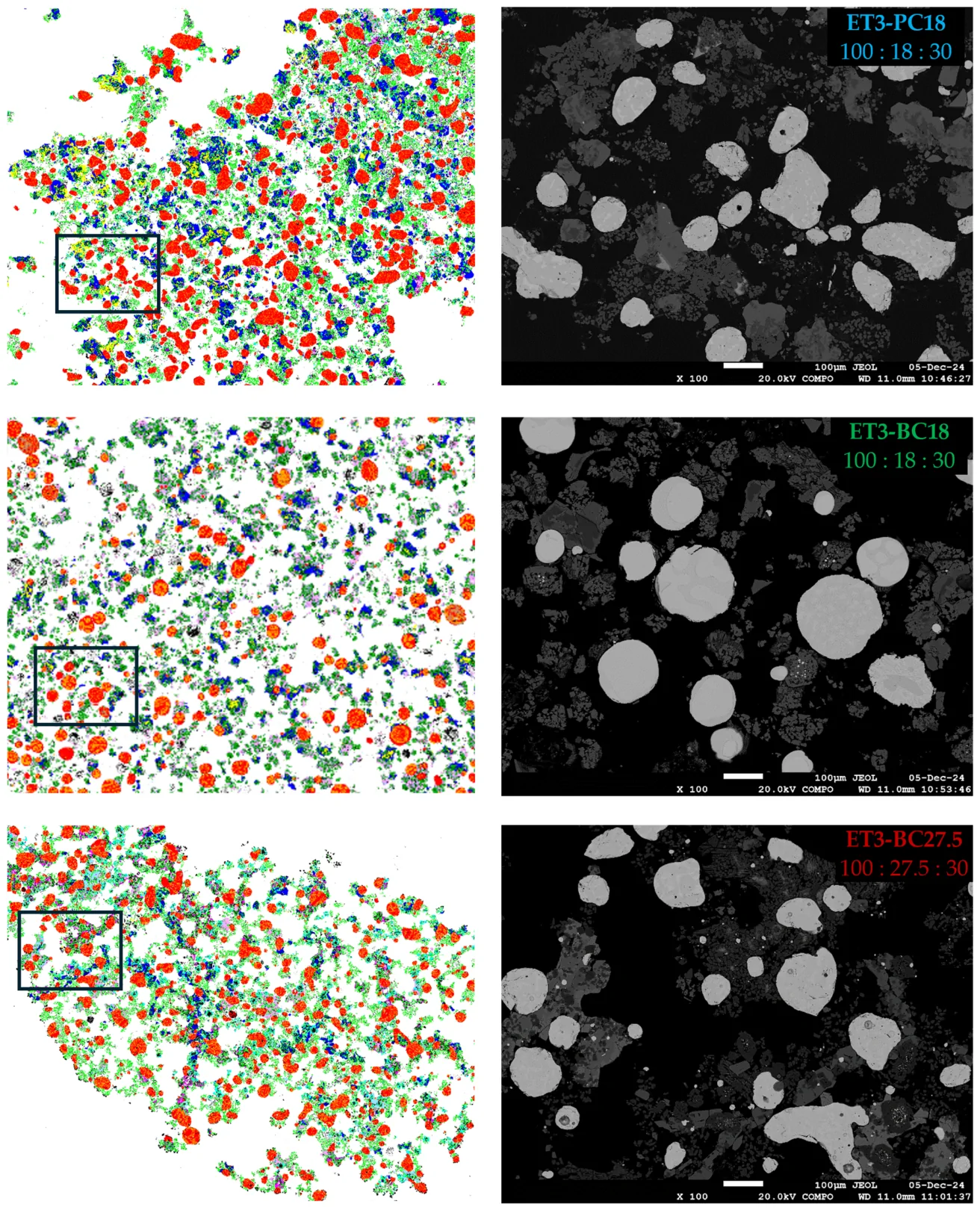
Representative backscattered electron (BSE) images of alloy and slag phases in reduced pellets (**right**). Slag phases appear in varying greyscale tones based on composition while alloy particles appear white. TIMA phase maps (**left**) show BSE image regions denoted by frames and phase colors corresponding to the legend in Figure 8.

**Figure 10:**
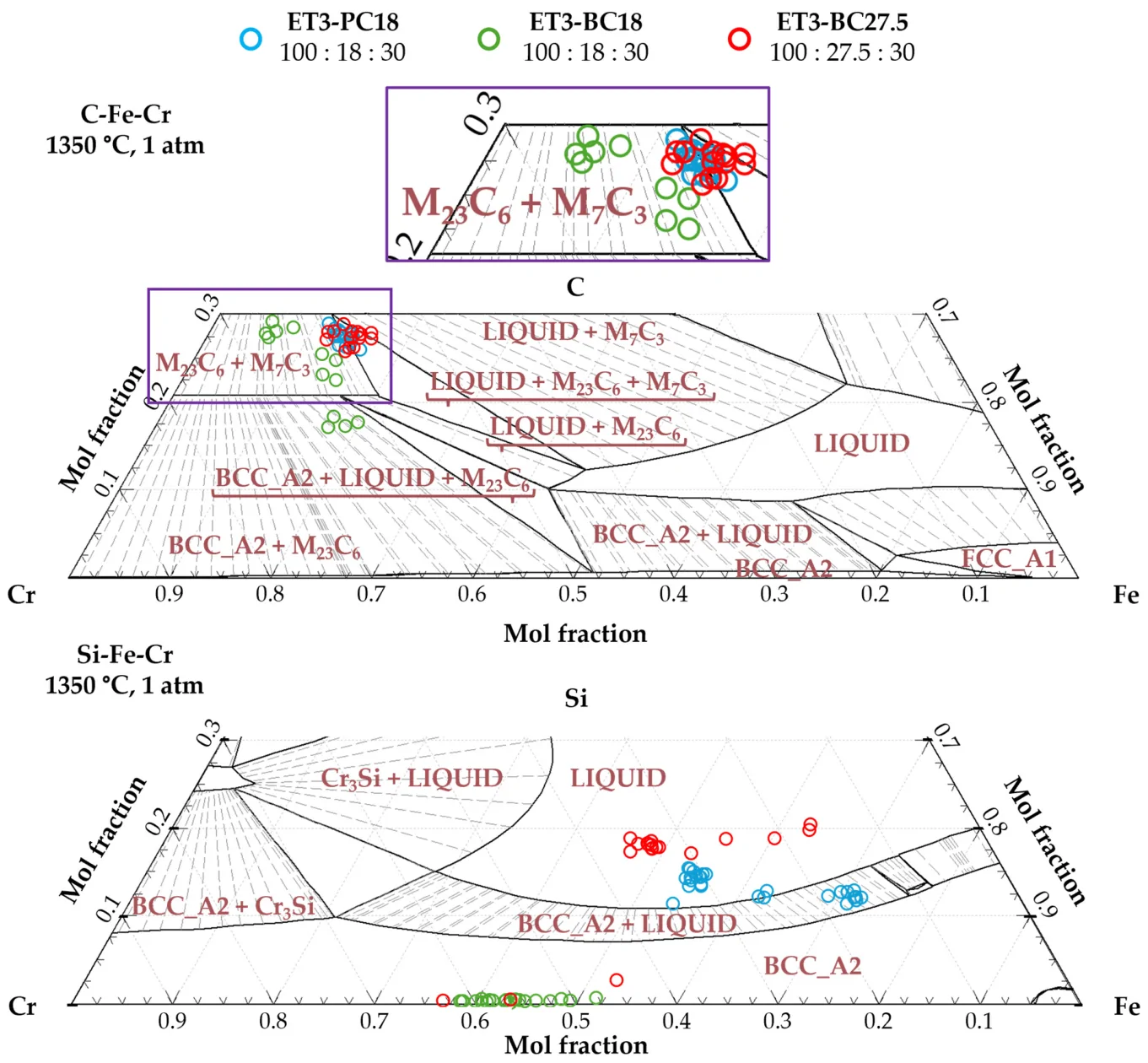
Ternary phase diagrams showing WDS-EPMA analyses of alloy phases plotted on C-Fe-Cr (for Si-free alloy phases) and Si-Cr-Fe (for C-free alloy phases) systems at 1350 °C, 1 atm, generated using FactsageTM [42].

**Figure 11:**
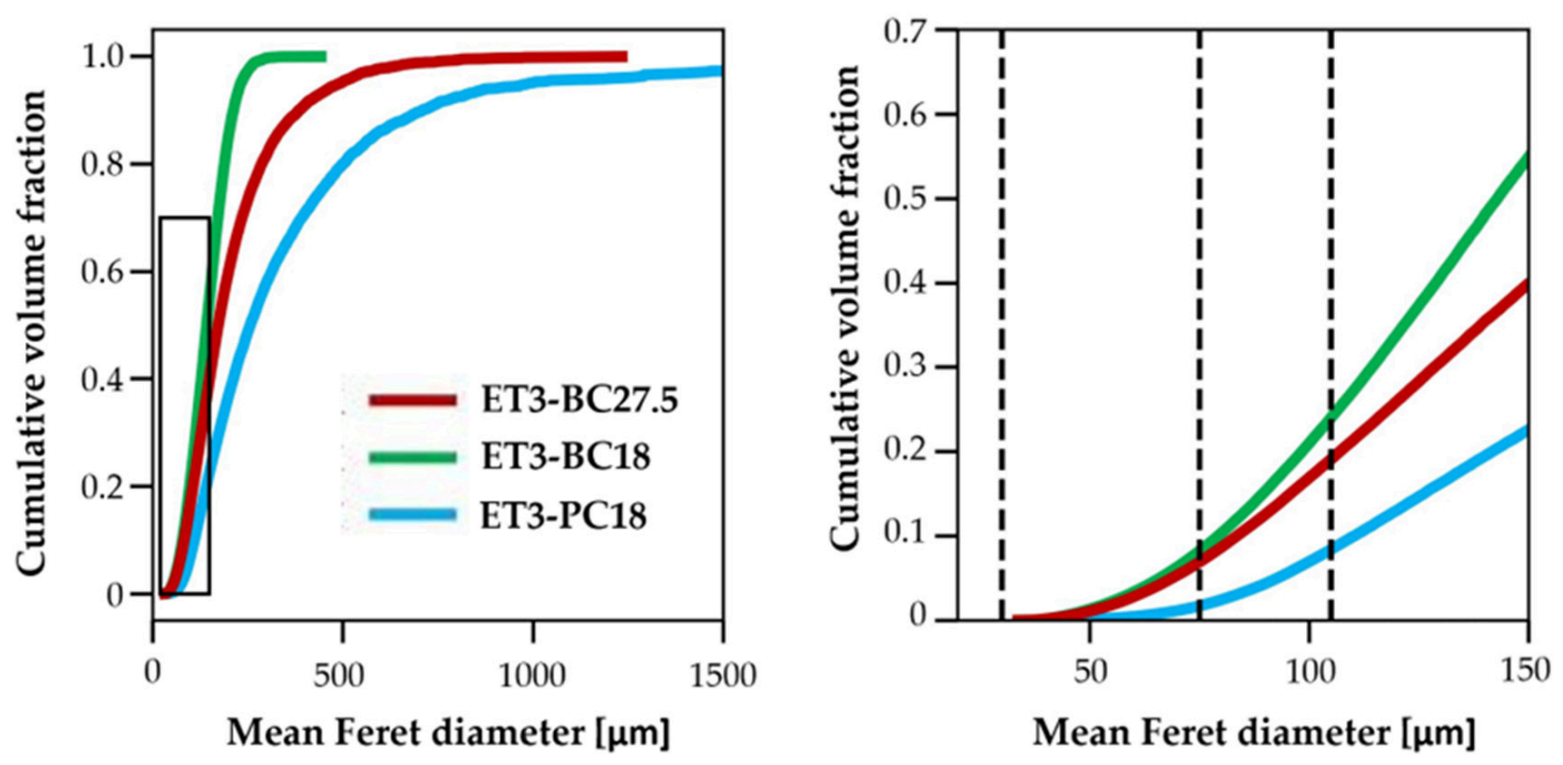
Alloy particle size distributions obtained from X-ray microtomography, with particle size defined as the mean Feret diameter of each particle. The y-axis shows cumulative volume fraction. The right-hand panel magnifies the region outlined by the black box, with dashed lines marking 20, 75, and 105 µm. Particle counts are 70,330 for ET3-PC18, 137,187 for ET3-BC18, and 155,992 for ET3-BC27.5.

**Figure 12:**
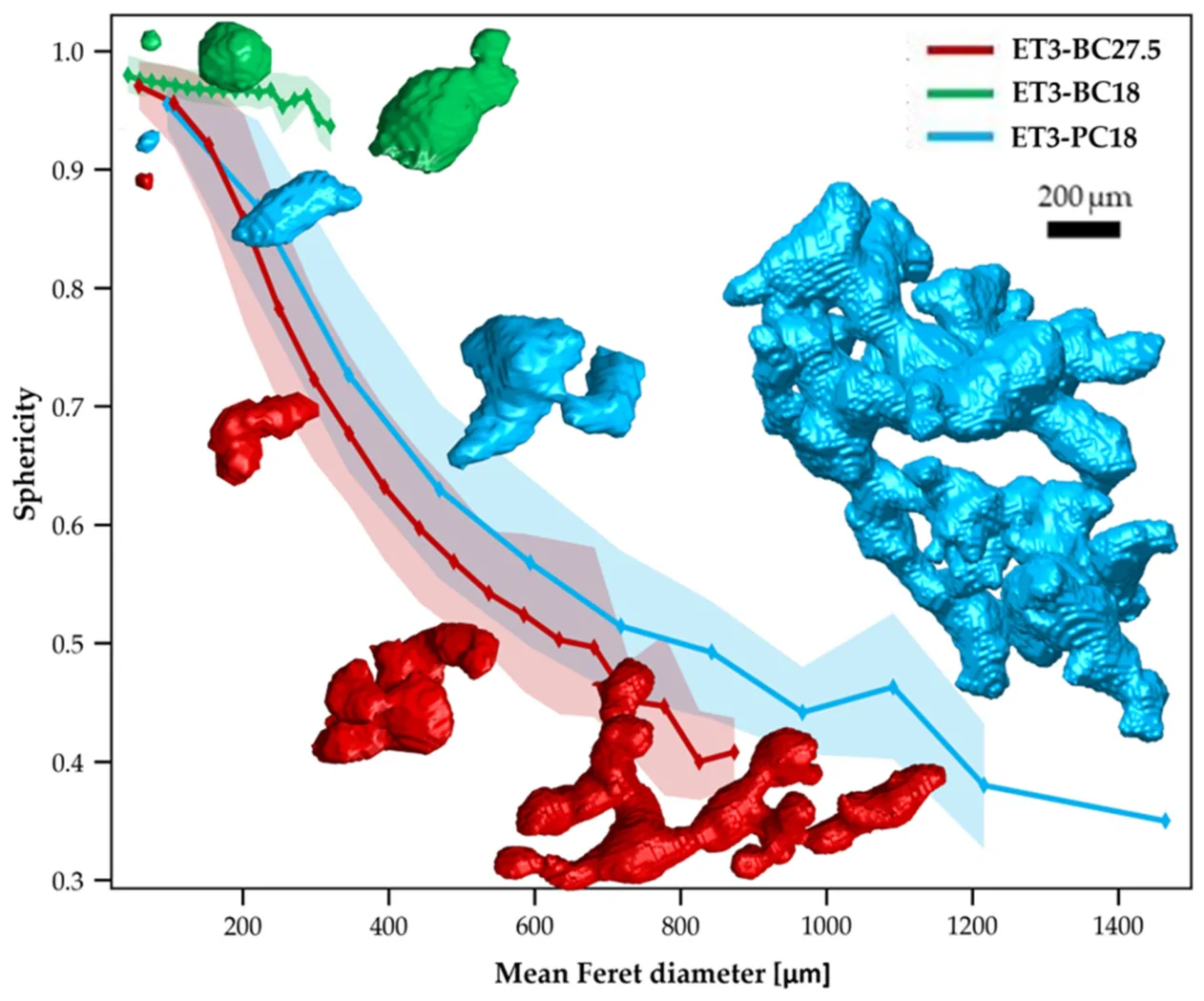
X-ray microtomography particle shape and size analysis, where size is the mean Feret diameter and shape is sphericity of each particle. Mean sphericity was calculated using a 25-window sliding-window method, with diamond markers indicating window centroids; the window half-width equals the centroid spacing. Colored bands show ±1 standard deviation of sphericity for particles within each window’s mean Feret diameter. Scaled particle renderings illustrate particle shape and morphology changes with Feret diameter.

Normalized CO fluxes (Figure 7) and phase proportions determined using TIMA (Figure 8) characterize reduction behavior and phase evolution. Representative BSE images and TIMA phase maps are shown in Figure 9. WDS-EPMA analysis of the alloy phases is overlain onto ternary phase diagrams and presented in Figure 10. The compositions of selected residual and slag phases, which were also determined using WDS-EPMA analysis, are shown in Table 4. Assay values for separated fractions are shown in Table 5. The particle size distributions and sphericities of alloy particles determined by X-ray microtomography are shown in Figure 11 and Figure 12.

The CO off-gas profiles in Figure 7 are generally consistent with those in Figure 3. ET3-BC18 and ET3-BC27.5 exhibit greater CO flux below the CaCl_2_ melting point (132 min) compared to ET3-PC18, although some of this may reflect CO_2_ interference. The main CO peak onset occurs at a comparable temperature of approximately 1000 °C for all experiments. Between 190 and 210 min, the slope of CO in the pre-peak segment is greater for ET3-BC18 and ET3-BC27.5 than for ET3-PC18. Peak CO flux occurs at elapsed times of 224 min, 228 min, and 232 min for ET3-BC27.5, ET3-BC18, and ET3-PC18, respectively. The normalized CO fluxes at the end of the high temperature dwell are similar across tests (0.0002 g_CO_·g_ore_^−1^·min^−1^).

Phase quantification by area using TIMA (Figure 8) shows that alloy phases constitute 34.0% and 34.6% for ET3-PC18 and ET3-BC27.5, respectively, but only 18.6% for ET3-BC18. Si-bearing alloy phases were identified in ET3-PC18 and ET3-BC27.5 but not in ET3-BC18. Slag phases are dominated by spinel group minerals. Magnesium-aluminate spinel accounts for approximately 27.0% of ET3-PC18 and ET3-BC27.5, whereas ET3-BC18 contains 34.1% magnesium-chromite. Olivine group minerals (monticellite and forsterite) are 17.2%, 12.4%, and 3.7% for ET3-PC18, ET3-BC18, and ET3-BC27.5, respectively. Cl-bearing silicates (including wadalite) are present in all samples and dominant in ET3-BC18 (19.8%) and ET3-BC27.5 (7.6%) but minor in ET3-PC18 (1.4%). Minor MgO occurs in ET3-PC18 and ET3-BC27.5 but not in ET3-BC18. Unclassified phase ranges are reasonable for TIMA analysis and comparable across all samples (14.5–15.9%).

Ternary phase diagrams (Figure 10) show alloy phase compositions determined using WDS-EPMA analysis for the C-Fe-Cr and Si-Fe-Cr systems. All samples contained M_7_C_3_ and BCC phases; ET3-BC18 additionally exhibited M_23_C_6_. ET3-BC18 showed the greatest variability in alloy phase composition, with Cr:Fe ratios ranging from 3.0–8.1 in M_23_C_6_-M_7_C_3_-BCC phase regions and 1.0–1.5 in BCC phases. Alloy compositions for ET3-PC18 and ET3-BC27.5 were generally comparable, consisting of M_7_C_3_ phases with Cr:Fe ratios of ~4.5:1, along with BCC and liquid phases containing no carbon and up to 20 mol% Si. ET3-BC27.5 exhibited slightly higher Si contents than ET3-PC18, and Cr:Fe ratios for BCC-liquid phases ranged from 0.2–0.7.

WDS-EPMA analyses of measured spinel, silicate, and olivine phases were averaged and are summarized in Table 4. Spinels in ET3-PC18 and ET3-BC27.5 were magnesium-aluminate with less than 1.03 wt% Cr_2_O_3_, whereas ET3-BC18 contained magnesium-chromite with 33.2 wt% Cr_2_O_3_ and correspondingly less Al_2_O_3_. The monticellite (CaMgSiO_4_) compositions of ET3-PC18 and ET3-BC18 were the most similar, while ET3-BC27.5’s monticellite contained comparatively less MgO and more CaO. For ET3-BC27.5, monticellite is therefore the major olivine phase and likely associated with merwinite (Ca_3_MgSi_2_O_8_), which is adjacent to the monticellite field in the CaO-MgO-SiO_2_ ternary phase diagram [18]. Forsterite (Mg_2_SiO_4_) phases in ET3-PC18 and ET3-BC18 were comparable but contained < 5.40% CaO, although ET3-BC18 contained more CrO (1.02 wt% vs. 0.06 wt%). Rondorfite (Ca_8_Mg(SiO_4_)_4_Cl_2_) in ET3-BC27.5 contained 9.20 wt% Cl. No other silicates (Cl or non-Cl-bearing) were identified in the areas examined by WDS-EPMA for ET3-PC-18 and ET3-BC18.

Assayed compositions (ICP-OES) of separated alloy concentrates, tailings, and fines fractions are presented in Table 5. No mass losses were observed for ET3-PC18 and ET3-BC18 during mineral separation tests of the reduced pellets. However, ET3-BC27.5 exhibited a 19.6 wt% loss. The amount of concentrate obtained was highest for ET3-PC18 (58.9 wt%) and lowest for ET3-BC27.5 (41.3 wt%). Tailings were most abundant in ET3-BC18 (40.9 wt%) and least in ET3-BC27.5 (8.2 wt%). Head sample compositions for ET3-PC18 and ET3-BC27.5 were similar, whereas ET3-BC18 contained notably less Fe, Cr, and C, which was offset with a greater proportion of other constituents (O with minor Cl) and slightly higher Ca, Mg, and Al concentrations. Weighted sums across fractions were within 3 wt% of head values for all analytes except Cr, which deviated by up to 6 wt%.

The lost fraction of ET3-BC27.5 (19.6 wt%) is compositionally consistent with the +38 tailings fraction and is therefore attributed to a combination of tailings-type particles finer than 38 µm and water-soluble phases that were lost during the dewatering of the fines fraction. The comparatively low tails yield of ET3-BC27.5 relative to ET3-PC18, despite similar fixed carbon contents, supports this interpretation.

Component balances for Fe and Cr were calculated from the data in Table 5 using Equation (2), where *m* is the mass of each product stream, *x* is the elemental composition, and *i* denotes the C, T, and F fractions. H denotes the reduced pellet heads fraction. The component balance discrepancy, ε, was used to assess the impact of the unaccounted 19.6 wt% mass loss in ET3-BC27.5 on Fe and Cr recovery. A 10.3% deficit in Fe and Cr recovery was determined for ET3-BC27.5. However, component balance discrepancies of similar magnitude were also obtained for ET3-PC18 (−12.7%) and ET3-BC18 (+14.3%), despite no evident material losses in either test. As the ET3-BC27.5 deficit falls within this range, the apparent loss of Fe and Cr cannot be distinguished from experimental uncertainty in the mass and compositional measurements.

(2) $$m_{H}x_{H,Fe+Cr}-\sum_{i=C,T,F}m_{i}x_{i,Fe+Cr}=\varepsilon_{Fe+Cr}$$

Table 5 shows that the alloy concentrates from ET3-PC18 and ET3-BC27.5 were predominantly Cr and Fe, with Cr:Fe ratios of ~2.1:1, consistent with head samples and the ore’s composition indicating near complete metallization. The concentrate compositions recalculated in terms of Cr-Fe-C-Si for ET3-PC18 and ET3-BC27.5 are 61.35%–29.40%–6.90%–2.35% and 60.85%–28.50%–6.22%–4.43%, respectively. These compositions contain amounts of C and Si which would be positioned a modest distance away from the common Fe-Cr edge of a Cr-Fe-C-Si phase tetrahedron and would tend toward the M_7_C_3_ field on the C-Fe-Cr face of the tetrahedron to a slightly greater extent than the BCC fields on the Si-Fe-Cr face (Faces shown in Figure 10.) Application of the lever rule therefore indicates that M7C3 constitutes the majority alloy phase in ET3-PC18 and ET3-BC27.5. The ET3-BC18 concentrate also consisted mainly of Cr and Fe but had a much lower Cr:Fe ratio (1.65) and a reduced C content (1.45 wt% vs. 5.92 wt% and 6.54 wt%), suggesting that the amount of carbon reductant in the feed was insufficient and that the Cr metallization was incomplete. The concentrate composition for ET3-BC18 recalculated in terms of Cr-Fe-C-Si is 61.08%–37.05%–1.48%–0.39%. This composition contains low amounts of C and Si, which would approach the common Fe-Cr edge (where C and Si = 0) of a Cr-Fe-C-Si phase tetrahedron, and tend toward the BCC fields at 0 carbon and 0 Si conditions of the C-Fe-Cr and Si-Fe-Cr faces. This composition qualitatively indicates a higher proportion of BCC phases according to the lever rule for multiphase systems. Other constituents were <3.91 wt% for all samples, and Mg and Al were <1.5 wt%. All concentrates meet ISO 5448 specifications for High Carbon Ferrochrome [46].

Tails from ET3-PC18 contained the highest residual phase element compositions (Al, Ca, Mg, Si) and the lowest alloy phase element compositions (Cr, Fe, C) in comparison to the tails from the other ET3 tests. The Cr contents of the tails fractions were 3.08 wt%, 18.78 wt%, and 23.91 wt% for ET3-PC18, ET3-BC18, and ET3-BC27.5, respectively. The fines from ET3-PC18 also contained higher amounts of Al, Mg, and Si and lower amounts of Cr than the fines from the other ET3 tests. The Cr contents of the fines fractions were 9.91 wt%, 19.58 wt%, and 40.06 wt% for ET3-PC18, ET3-BC18, and ET3-BC27.5, respectively. The fractions with the highest proportions of other constituents (O with minor Cl) for each experiment were the tails in ET3-PC18 (40.97 wt%) and ET3-BC18 (39.00 wt%) and the calculated lost fraction in ET3-BC27.5 (46.56 wt%).

The cumulative volume fraction with size as determined by the Feret diameter in Figure 11 shows that alloy particles in ET3-PC18 are larger than those in ET3-BC27.5 at all equivalent cumulative volume fractions. The largest 20% of ET3-PC18 particles by volume exhibit a broad size distribution, ranging from 500 to over 1500 µm in mean Feret diameter. In contrast, the largest 20% of ET3-BC18 particles range from 190–443 µm and ET3-BC27.5 particles from 296–1233 µm. Alloy particles smaller than 75 µm account for approximately 7% of the total alloy volume in ET3-BC18 and ET3-BC27.5, but less than 2% in ET3-PC18.

Sphericity ψ is a computed measurement represented by the ratio of the surface area of a sphere with a given volume divided by the measured surface area and was calculated using ($\psi =\;\frac{\pi^{\frac{1}{3}}{\left(6V\right)}^{\frac{2}{3}}}{A})$, where V is volume and A is surface area. Alloy particles are well rounded, with comparable sphericity for ET3-PC18 and ET3-BC27.5 and greater in ET3-BC18. Sphericity trends (Figure 12) show that ET3-BC18 particles are the most spherical, with values exceeding 0.95 across almost all size ranges. For ET3-PC18 and ET3-BC27.5, sphericity exceeds 0.95 at Feret diameters below 106 µm then decreases drastically with increasing particle size, dropping below 0.4 for the largest particles. Below 106 µm, ET3-BC27.5 particles are slightly more spherical than ET3-PC18, whereas at larger sizes, ET3-BC27.5 particles are less spherical at a given size. The tomography results are consistent with BSE and TIMA imaging (Figure 9), which illustrate phase textures and spatial relationships in 2D.

#### 3.3.1. Impacts of Bio-Carbon as a Reductant on Reaction Kinetics

CO evolution profiles are used to compare the kinetic behavior of BC and PC under controlled test conditions. ET3-PC18 provides the reference behavior for a fixed mass of reductant, ET3-BC18 isolates the effect of replacing this mass directly with BC (and contains less fixed carbon), and ET3-BC27.5 represents the case where BC contains a comparable quantity of fixed carbon to ET3-PC18.

As noted in Section 3.1 and previous studies [18,20,21], following CaCl_2_ melting at 772 °C, carbothermic reduction of reducible oxides generates CO at temperatures above ~1100 °C. Figure 3 indicates that CO_2_ evolution is minor at and above this temperature relative to CO production. Accordingly, CO-only profiles (Figure 7) provide a reliable basis for qualitative kinetic comparison. The slope of the main CO-evolution event between 190–210 min increases in the order ET3-PC18 < ET3-BC18 < ET3-BC27.5, indicating progressively faster reduction with BC and with increasing BC content. Peak CO flux occurs the earliest for ET3-BC27.5 (224 min), closely followed by ET-BC18 (228 min) and ET3-PC18 (232 min), further confirming the kinetic enhancement associated with BC. The onset temperature of the major CO-generation event is nearly identical across all tests (Figure 3 and Figure 7), demonstrating that these rate differences arise from physical rather than compositional differences between the reductants.

At the end of the high-temperature dwell, CO fluxes converge to ~0.0002 g_CO_·g_ore_^−1^·min^−1^, indicating that all systems have reached near-complete reaction extent. This can either be due to the exhaustion of reducible oxides, fixed carbon, or both in the case of a stoichiometric system. Compared with the oxygen content (with minor Cl from slag phases) in the ET3-PC18 and ET3-BC27.5 head samples (14.2 ± 0.1 wt%), ET3-BC18 retains significantly more oxygen (and minor Cl) at 23.85 wt%, reflecting incomplete reduction due to insufficient fixed carbon. These results confirm that ET3-BC27.5 appropriately represents devolatilized BC with comparable fixed carbon to ET3-PC18, resulting in near-complete reduction of reducible oxides (stoichiometric or excess carbon).

The kinetic advantage of BC principally arises from its devolatilization, which increases its porosity and effective surface area [47], thereby accelerating gas-solid reactions under shrinking-core conditions [13,16,17]. Increasing BC addition further raises the total reactive surface area, accounting for the faster reduction observed in ET3-BC27.5 compared with ET3-BC18. Microtomography results (Figure 11) show that alloy particles smaller than the initial BC particle size (<75 µm) constitute ~7% of the total alloy volume in both ET3-BC18 and ET3-BC27.5, but less than 2% in ET3-PC18. This trend suggests that porosity generated in BC during devolatilization is diminished by alloy densification.

Collectively, these observations show a clear kinetic enhancement when BC replaces PC, primarily attributable to BC’s devolatilization and its resulting reactive microstructure. Quantification of this advantage using formal kinetic analysis methods is recommended for future work [48].

#### 3.3.2. Impacts of Bio-Carbon as a Reductant on Alloy Product Quality

To evaluate whether DRC-derived alloy particles could substitute for conventional FeCr ingots in stainless steel production, both alloy chemistry and particle morphology must be assessed. In the present study, although further liberation optimization could reduce Ca, Al, and Mg entrainment in the concentrates, the impurity levels obtained are already sufficiently low to meet ISO 5448 specifications [46], and demonstrate the technical feasibility of producing FeCr via CaCl_2_-assisted DRC using BC as the reductant.

Alloy composition and concentrate chemistry vary consistently with BC addition, reflecting a shift from carbon-limited to carbon-sufficient reduction. WDS-EPMA compositions plotted on FactSage Fe-Cr-C ternaries (Figure 10) indicate that the alloys formed under carbon-sufficient conditions are made of M_7_C_3_ carbides and BCC Fe-Cr.

At low BC addition (ET3-BC18), Fe is reduced. However, carbon is sub-stoichiometric for complete reduction of Cr oxides. Carbon is exhausted before all Cr is reduced and the concentrate therefore contains less C and exhibits a lower Cr:Fe ratio than the ore (1.65 vs. 2.14 Table 1 and Table 5), consistent with incomplete Cr metallization. This variation is attributable to the contribution of lower carbon M_23_C_6_ and BCC Fe-Cr phases, which were identified using WDS-EPMA in addition to M_7_C_3_ phases for ET3-BC18 (Figure 10). The M_23_C_6_ and BCC Fe-Cr phases observed in ET3-BC18 likely formed only after the complete consumption of BC in carbothermic reactions that produced M_7_C_3_, as these phases are absent for ET3-PC18 and ET3-BC27.5, as seen in Figure 10. Coexistence of M_7_C_3_ and M_23_C_6_ in relatively low-carbon Fe-Cr-C alloys has been reported previously [49,50], although the proposed formation pathways require the presence of liquid alloy phases and differ. Wieczerzak et al. contend that early M_7_C_3_ precipitation from a liquid alloy phase depletes Cr and C and promotes subsequent M_23_C_6_ formation, whereas Roshchin et al. suggest that carbon dissolved in slag can drive later-stage reduction at reductant/slag interfaces, producing a carbon-lean alloy that dilutes a carbide-rich melt. Although FactSage does not predict a liquid field for the measured ET3-BC18 points, several measured compositions plot near liquid-containing phase regions (Figure 10), and their narrow phase boundaries in the Fe-Cr-C system remain sensitive to experimental conditions and are still debated [49,51,52,53,54,55,56].

With increased BC (ET3-BC27.5), carbon becomes sufficient (or excess) to support near complete Cr reduction. In this regime, WDS-EPMA data indicate predominantly M_7_C_3_-type FeCr (Figure 10), and TIMA shows a higher proportion of Cr-rich alloy relative to the carbon-limited case ET3-BC18 (Figure 8). Thus, a higher BC content improves Cr metallization. Cr contents in the tails and fines of ET3-BC27.5 are, however, elevated compared to ET3-PC18 (T = 23.91 wt% Cr and F = 40.06 wt% Cr vs. T = 3.08 wt% Cr 9.91 wt% Cr), indicating an incomplete recovery of FeCr in the ET3-BC27.5 concentrate.

A higher BC (or other reductant) addition also increases impurity pickup, most notably Si. Silicon is detected in alloys for ET3-BC27.5 (and ET3-PC18) but is minimized at a lower carbon input in ET3-BC18. This trend is consistent with increasingly reducing conditions at higher carbon levels, driven by elevated CO/CO_2_ ratios that lower oxygen fugacity. The resulting decrease in oxygen potential favors reduction of silica (SiO_2_) to Si, leading to increased incorporation into the alloy. The absence of unreacted cores in Figure 5 at ore:BC ratios of 100:25 and higher may therefore be attributable to this mechanism, whereby excess carbon for Fe and Cr metallization participates in silica reduction. While the Ellingham diagram for SiO_2_ reduction indicates that direct reduction by solid carbon is thermodynamically unfavorable at 1350 °C under standard conditions [57], experimental studies show that Si can be reduced in Fe-based melts containing dissolved carbon [58]. This apparent discrepancy arises from the higher effective activity of carbon in solution [59], the reduced activity of Si in the alloy, and locally lower oxygen potentials at the metal-slag interface, which together shift the equilibrium to favor SiO_2_ reduction. Gas-phase transport pathways may also contribute: Hagashi et al. [60] propose a partial reduction of SiO_2_ to SiO, followed by SiO transport and reduction at Fe-C interfaces. Under DRC conditions, the highly effective slag-metal interfacial area made up of dispersed particles surrounded by slag and flux plausibly enhances such interfacial transfer relative to a single planar interface under smelting conditions. Related particulate reduction studies report Si-bearing products, including silicides, after high temperature reduction of chromite-silicate mixtures [61], and SiO generation has been discussed for silica-bearing slag and carbothermic systems [60,62]. The occurrence of Si in FeCr produced at 1350 °C is therefore consistent with previous observations. However, further investigation is required to identify the underlying mechanism.

Reductant properties also govern alloy morphology. Because BC undergoes devolatilization (detailed in Section 3.1), its carbon density is lower than that of PC, which directly influences the resulting alloy size distribution after metallization. Integrating previous research [18,19,20], tomography, TIMA phase areas, and WDS-EPMA results suggests three morphological stages. (i) Alloy rims form while particles largely retain their reductant geometry because the devolatilized BC is predominantly graphitic and remains solid at the temperatures used. The rims of neighboring particles can also sinter together, resulting in larger irregular aggregates, as shown by previous research (e.g., [18] Figure 2c within). (ii) Once the carbon cores are consumed, particles round and densify to reduce surface energy. (iii) Neighboring particles coalesce during rounding, producing particles with diameters several times greater than the initial reductant size (Figure 12). Isolated particles do not aggregate.

Across all tests, alloys are largely free of unreacted reductant cores, and some can have particle diameters larger than the initial carbon size distribution (Figure 11). Tomography evidence of necked contacts and large irregular aggregates in ET3-PC18 and ET3-BC27.5 (Figure 12) therefore supports sintering of neighboring particles as the mechanism to create particles larger than the reductant particles’ starting size [63]. This is emphasized by a sharp decrease in sphericity in ET3-PC18 and ET3-BC27.5 alloys above 106 µm. ET3-BC18 alloys larger than 106 µm exhibit the highest sphericity relative to ET3-PC18 and ET3-BC27.5 (Figure 12). This behavior is attributed to reduced interparticle contact and increased densification.

These morphology–chemistry relationships can be critical to DRC optimization. The addition of stoichiometric carbon is required for Fe and Cr metallization, but replacing PC with BC reduces pellet volumetric productivity (less processed ore per pellet volume), lowering throughput. Conversely, the use of BC may accelerate reduction kinetics.

#### 3.3.3. Impacts of Bio-Carbon as a Reductant on Slag Chemistry and Residual Phase Chemistry

As mentioned previously, DRC slag can be considered as a salable coproduct with FeCr, and is dominated by refractory phases (spinels, olivines, and MgO), as well as meaningful amounts of silicates and glassy interstitial phases. Minor amounts of other oxides, carbonates, and sulfides may also be present [18,19,20,21]. The present results allow assessment of how BC and PC affect slag phase compositions and their proportions.

During DRC (with either BC or PC as the reductant), Cr is incongruently dissolved from the spinel, which becomes progressively Mg enriched. Toward the final stages of reduction with continued Cr release, excess Mg is partitioned to the melt and incorporated into Ca-silicate slags. Increasing BC addition (ET3-BC18 → ET3-BC27.5) reduces the Cr_2_O_3_ content in residual spinel from 33.15 to 0.69 wt% (WDS-EPMA, Table 4), consistent with TIMA classifications (Figure 8) which show the spinel phase evolving from Mg-chromite (ET3-BC18) to Mg-aluminate spinel (ET3-BC27.5). Because BC has lower fixed carbon, a higher BC-to-ore mass ratio is required to achieve the same metallization as PC.

Silicate phases in the slag were classified in all ET3 samples by TIMA (Figure 8), though only ET3-BC27.5 yielded grains that are large enough for WDS-EPMA analysis. The silicates in ET3-PC18 and ET3-BC18 appear small, porous, and fragmented (Figure 9), hindering quantitative microprobe analysis. Consistent with previous work, blast furnace type slags and Ca-silicate slags incorporating halide-bearing phases are expected under these conditions [18,64]. When Ca and Si are abundant, rondorfite (Ca_8_Mg(SiO_4_)_4_Cl_2_) is favored in Mg- and Cl-rich environments, while wadalite ((Ca,Mg)_6_(Al,Fe^3+^)_4_((Si,Al)O_4_)_3_O_4_Cl_3_) occurs where Al and Cl are comparatively enriched. Rondorfite is confirmed using WDS-EPMA in ET3-BC27.5 (Table 4), and Cl-bearing silicates were classified using TIMA across all samples (Figure 8).

The olivine minerals monticellite and forsterite were identified using TIMA for all ET3 tests (Figure 8) and were measured using WDS-EPMA except for forsterite belonging to ET3-BC27.5 (Table 4). The monticellite in ET3-BC27.5 contains more CaO (40.68 wt%) and less MgO (22.54 wt%) and SiO_2_ (37.55 wt%) compared with ET3-PC18 and ET3-BC18 (28.83 ± 0.08 wt% MgO, 33.22 ± 0.38 wt% CaO, 38.31 ± 0.03 wt% SiO_2_ Table 4), indicating an average mixture composition of monticellite and merwinite (Ca_3_Mg(SiO_4_)_2_). Forsterite compositions in ET3-PC18 and ET3-BC18 are similar, though ET3-BC18, conducted under carbon-deficient conditions relative to those required for complete metallization of Cr and Fe, exhibited elevated Cr_2_O_3_ (1.02 wt% vs. 0.06 wt%). Significant influence from sub-surface micro-inclusions of spinel is precluded by the low measured amounts of Al_2_O_3_ in this forsterite (0.07%). This is consistent with our previous observations made for sub-stoichiometric carbon experiments contemplating that Cr dissolved into molten slag/salt is reduced to 2^+^ [18]. This is also consistent with other studies showing that under reducing conditions and at temperatures > 1100 °C, incorporation of Cr^2+^ into olivine is feasible [43,44,45].

ET3-PC18 and ET3-BC27.5 exhibit comparable areal proportions of alloy and spinel phases based on TIMA analysis (Figure 8) yet differ in the distribution of olivine and non-olivine slag phases. The former contains 17.2% olivine and 6.4% non-olivine slag, whereas the latter contains 3.7% olivine and 18.7% non-olivine slag. Within the non-olivine slag fraction, MgO contents are of the same order (3.2% and 4.9%), while Cl-bearing slag phases are more abundant for ET3-BC27.5 (7.6% vs. 1.4%). These differences suggest that in some circumstances, reductant type can influence the partitioning of non-alloying elements among slag phases, in addition to its role in Fe and Cr reduction and metallization.

## 4. Conclusions

This work addresses a key knowledge gap relevant to assessing the use of biochar in CaCl_2_-assisted direct reduction of chromite (DRC) as a lower-carbon alternative to conventional reductants. The following main conclusions are drawn:

1. Bio-carbon (BC) enhances reduction kinetics relative to petroleum coke (PC). The rate of CO flux increased more rapidly for BC-containing pellets during the early stages of reduction, and peak CO flux was reached earlier than for PC-containing pellets at comparable fixed carbon contents. This behavior is attributed to BC having a more porous structure, which increases its effective reactive surface area. Because BC contains less fixed carbon than PC, higher BC additions (i.e., more reductant) are required to achieve similar extents of Cr reduction and metallization. This will reduce the proportion of ore processed as mixed pellets on a volumetric basis.
2. Micro-CT analysis in this work revealed three stages of morphological changes to alloy particles, which builds on previous findings from 2D analysis using polished sections [18,19,20,21]. These stages are: (i) initial alloy rim formation on/around reductant particles and neck formation of neighboring particles, forming irregular aggregates, (ii) rounding and densification once carbon is consumed, and (iii) coalescence producing round particles larger than their initial carbon size. BC promotes finer alloy populations due to its devolatilization at temperatures below 1000 °C and subsequent alloy densification. These phenomena and their interrelationships inform optimized processing conditions for efficient alloy recovery, since larger, more spherical particles are more readily recovered from reduced pellets.
3. WDS-EPMA compositions plotted on FactSage ternary phase diagrams confirm liquid-containing phase fields for both reductant types (ET3-PC18 and ET3-BC27.5). Liquid-influenced behavior contributes to particle rounding, coalescence, and the morphological trends observed which also inform the design of optimized processing conditions for efficient alloy recovery.
4. TIMA analysis demonstrates that BC as a reductant promotes the formation of non-olivine slag phases relative to olivine and increases the proportion of Cl-bearing slag phases. These observations indicate that reductant type can influence the partitioning behavior of non-alloying elements within the slag phases. This modified phase distribution has implications for the refractoriness and value of DRC slag.

## Figures and Tables

**Table 1:** Composition of the ore determined by XRF (wt%).

Al_2_O_3_	CaO	Cr_2_O_3_	Fe_2_O_3_(T)	MgO	MnO	NiO	P_2_O_5_	SiO_2_	TiO_2_	V_2_O_5_	LOI	Total
12.63	0.33	42.16	19.28	14.78	0.19	0.2	<0.01	7.68	0.36	0.15	2.41	100.2

**Table 2:** Proximate and ultimate analyses of PC and BC were performed at the CanmetENERGY characterization lab. Results are shown on a dry, ash free basis, <DL = below detection limit. The proximate analysis utilized methods ASTM D7582 [26] and ISO 562. [27] The ultimate analysis utilized methods ASTM D4239 [28] and D5373 [29], excluding oxygen, which was determined by difference.

Properties		PC	BC
**Proximate analysis** **[wt%]**	Moisture	0	0
	Volatile matter	0.5	38.4
	Fixed Carbon	99.5	61.6
	Ash content	0	0
	SUBTOTAL	100.00	100.00
**Ultimate analysis** **[wt%]**	Carbon	95.80	81.50
	Hydrogen	0.30	3.08
	Nitrogen	1.41	0.73
	Sulfur	2.08	<DL
	Oxygen	0.45	14.77
	SUBTOTAL	100.04	100.08

**Table 3:** Experimental conditions and design considerations of furnace tests. Feed compositions are shown as mass proportions of ore:reductant:flux.

Experiment ID		Feed	Reductant Type	Reductant Size Fraction [µm]	Dried Pellet Mass [mg]	Experimental Design Consideration
**TG1: Thermogravimetric**						
	TG1-PC18	68:12:20	PC	−106 + 75	213.6	Control; previously optimized conditions for Fe/Cr metallization using PC as a reductant.
	TG1-BC18	68:12:20	BC	−106 + 75	207.5	BC driven Fe/Cr metallization under sub-stoichiometric conditions.
**TG2: Thermogravimetric**						
	TG2-BC15	69:10:21	BC	−75	208.0	Limited reduction of Cr_2_O_3_ and Fe/Cr metallization.
	TG2-BC20	67:13:20	BC	−75	195.2	
	TG2-BC22.5	66:15:20	BC	−75	194.9	Optimization of BC proportion to minimize residual Cr_2_O_3_ in spinel and maximize Fe/Cr metallization.
	TG2-BC25	65:16:19	BC	−75	197.5	
	TG2-BC27.5	63:17:19	BC	−75	195.9	
	TG2-BC30	63:19:19	BC	−75	213.2	BC driven Fe/Cr metallization under super-stoichiometric conditions.
**ET3: Electric Tube Furnace**						
	ET3-PC18	68:12:20	PC	−106 + 75	6732.5	Control; previously optimized conditions for Fe/Cr metallization using PC as a reductant.
	ET3-BC18	68:12:20	BC	−106 + 75	6624.1	BC driven Fe/Cr metallization under sub-stoichiometric conditions.
	ET3-BC27.5	63:17:19	BC	−106 + 75	5081.9	Optimized BC proportion for Fe/Cr metallization informed by TG2 tests.

**Table 4:** Averaged compositions of slag phases (wt%) measured using WDS-EPMA. Number of analyses shown in brackets, <DL below detection limit. Cr in slag phases like olivine is reported as 2^+^ based on our earlier findings [18,43,44,45].

Phase		SiO_2_	Al_2_O_3_	Cr_2_O_3_	CrO *	FeO	CaO	MgO	Cl	Total
**ET3-PC18**										
Spinel	(20)	2.32	64.96	1.03		0.04	0.12	32.29	<DL	100.76
Monticellite	(17)	38.33	0.14		0.08	0.03	33.59	28.90	<DL	101.07
Forsterite	(16)	40.93	0.34		0.06	<DL	5.40	54.11	<DL	100.84
**ET3-BC18**										
Spinel	(20)	0.13	38.63	33.15		0.06	0.27	26.82	0.05	99.11
Monticellite	(17)	38.28	0.07		1.15	<DL	32.84	28.75	<DL	101.09
Forsterite	(16)	41.18	0.26		1.02	<DL	4.11	54.60	<DL	101.17
**ET3-BC27.5**										
Spinel	(17)	0.16	69.04	0.69		0.05	0.15	31.02	<DL	101.11
Monticellite	(16)	37.55	0.12		0.15	0.08	40.68	22.54	<DL	101.12
Rondorfite	(14)	30.45	0.60		0.12	0.05	57.78	4.69	9.20	100.81

**Table 5:** Mineral separation test results showing fractions (wt%) of concentrate, tails, and fines and analyte compositions (wt%) from ICP-OES assays. H = Reduced pellet Heads, C = Concentrates (> 38 µm), T = Tailings (> 38 µm), F = Fines (–38 µm), with numbers in brackets indicating mass percentages of the fractions. Concentrate fractions represent alloys, whereas the tails fractions represent slag components. Other constituents are mainly O with minor Cl from slag phases based on ore assay composition and use of CaCl_2_ as flux. ΣC,T,F represents the weighted sum of each analyte across all fractions.

		Al	Ca	Cr	Fe	Mg	Si	C Total	Other Constituents	Cr:Fe
**ET3-PC18**										
	Head H	4.63	3.55	42.31	20.32	5.82	4.35	4.83	14.19	2.08
	+38 C (58.9%)	0.36	0.39	58.13	27.85	0.59	2.23	6.54	3.91	2.09
	+38 T (20.5%)	14.50	9.80	3.08	1.56	20.02	9.52	0.55	40.97	1.97
	−38 F (20.7%)	20.16	4.39	9.91	5.11	16.00	5.89	1.26	37.28	1.94
	ΣC,T,F (100.1%)	7.36	3.15	36.92	17.78	7.76	4.48	4.22	18.42	2.08
**ET3-BC18**										
	Head H	7.47	4.40	34.63	16.73	7.79	4.30	0.83	23.85	2.07
	+38 C (49.4%)	0.10	0.43	59.73	36.23	0.14	0.38	1.45	1.54	1.65
	+38 T (40.9%)	13.31	5.65	18.78	3.17	14.30	5.32	0.47	39.00	5.92
	−38 F (9.8%)	13.67	4.51	19.58	4.19	13.59	4.46	3.55	36.45	4.67
	ΣC,T,F (100.1%)	6.83	2.97	39.11	19.60	7.25	2.80	1.26	20.28	2.00
**ET3-BC27.5**										
	Head H	4.91	2.99	42.69	19.83	6.38	4.15	4.84	14.21	2.15
	+38 C (41.3%)	0.66	0.85	57.88	27.11	1.51	4.21	5.92	1.86	2.14
	+38 T (8.2%)	7.91	6.49	23.91	11.29	15.10	4.57	4.16	26.57	2.12
	−38 F (30.9%)	9.96	3.98	40.06	18.53	11.41	4.80	4.34	6.92	2.16
	ΣC,T,F (80.4%)	4.00	2.11	38.24	17.85	5.39	3.60	4.13	5.09	2.14
	Calc Lost Fraction (19.6%)	4.65	4.47	22.69	10.11	5.06	2.82	3.64	46.56	2.24

## Data Availability

The original contributions presented in this study are included in the article. Further inquiries can be directed to the corresponding author.
